# Task shifting – Ghana's community mental health workers’ experiences and perceptions of their roles and scope of practice

**DOI:** 10.3402/gha.v8.28955

**Published:** 2015-10-09

**Authors:** Vincent I. O. Agyapong, Akwasi Osei, Conor K. Farren, Eilish McAuliffe

**Affiliations:** 1 Department of Psychiatry, Faculty of Medicine and Dentistry, University of Alberta, Edmonton, AB, Canada; 2 Department of Behavioural Sciences, Kwame Nkrumah University of Science and Technology, Kumasi, Ghana; 3 Centre for Global Health, Trinity College Dublin, University of Dublin, Dublin, Ireland; 4 Ghana Mental Health Authority, Accra, Ghana; 5 Accra Psychiatric Hospital, Accra, Ghana; 6 Department of Psychiatry, Trinity College Dublin, University of Dublin, Dublin, Ireland; 7 School of Nursing, Midwifery and Health Systems, University College Dublin, Dublin, Ireland

**Keywords:** community mental health workers, role, scope of work, mental health

## Abstract

**Background:**

Because of the absence of adequate numbers of psychiatrists, the bulk of mental health care at the community level in Ghana is provided by community mental health workers (CMHWs).

**Objective:**

To examine the role and scope of practice of CMHWs in Ghana from their own perspectives and to make recommendations to improve the care they provide.

**Design:**

We conducted a cross-sectional survey of 164 CMHWs from all the 10 administrative regions of Ghana, comprising 71 (43.3%) community psychiatric nurses (CPNs), 19 (11.6%) clinical psychiatric officers (CPOs), and 74 (45.1%) community mental health officers (CMHOs).

**Results:**

Overall, only 39 (23.8%) CMHWs worked closely with psychiatrists, 64 (39%) worked closely with social workers, 46 (28%) worked closely with psychologists and 13 (7.9%) worked closely with occupational therapists. A lower proportion of CMHOs worked closely with psychiatrists, psychologists, and social workers compared with CPOs and CPNs. There was no significant difference in the proportion of the different CMHW types who expressed confidence in their ability to diagnose any of the commonly named mental health conditions except personality disorders. However, a lower proportion of CMHOs than CPOs and CPNs expressed confidence in their ability to treat all the disorders. The CMHWs ranked schizophrenia as the most frequently treated mental health condition and there was no statistically significant difference in the reported frequency with which the three groups of CMHWs treated any of the mental health conditions.

**Conclusions:**

Mental health policy makers and coordinators need to thoroughly review the training curriculum and also evaluate the job descriptions of all CMHWs in Ghana to ensure that they are consistent with the demands and health-care needs of patients they care for in their communities. For example, as CMHOs and CPNs prescribe medication even though they are not expected to do so, it may be worth exploring the merits of including the prescription of common psychotropic medication in their training curriculum and job descriptions.

One of the cornerstones of primary health-care reforms advocated by the World Health Organization (WHO) in the 2008 World Health Report is achieving universal health coverage and reducing health inequalities (1). However, the severe shortage and imbalanced distribution of a trained health workforce poses a serious threat to achieving universal health coverage at the required scale and time frame (2). The critical shortages in human resources for health constitute one of the key barriers for accessing quality health care, and this is especially true in rural communities in Africa (3). The scarcity of mental health professionals, in particular, places specialized psychiatric care out of reach of most people in low- and middle-income countries (LAMICs) (4, 5), especially in the lowest income countries and in rural/low-income regions within countries (6). Mental health care relies heavily on trained workers, rather than on technology or tools (7), which means a shortage of trained mental health professionals, resulting in significant unmet mental health-care needs among populations. In a study of 58 LAMICs, Bruckner et al. (8) reported that the shortage in the mental health workforce amounts to about 239,000 workers, including 11,000 psychiatrists, 128,000 nurses in mental health settings, and 100,000 psychosocial care providers. The WHO estimates that 1.18 million additional mental health workers are needed to close the mental health treatment gap in LAMICs (9). Scaling up of mental health services that are safe, effective, and culturally sound presents significant challenges in a situation of ongoing poverty and multiple competing needs (10). Traditional and religious healers are known to be the primary sources of treatment for mental health problems in many LAMICs. However, no systematic data are available in LAMICs regarding the safety and efficacy of treatment of mental health problems by traditional or religious healers (11–14).

Thus, notwithstanding the visible burden, the field of global mental health can also be characterized by its inordinate resource gaps resulting in tragically high and persistent unmet needs (15). This resource gap calls for innovative and radical approaches to address the unmet needs. Despite the calls for increased attention to this issue, little research has focused on how to build the mental health workforce, particularly in Africa (16).

Mental health care in Ghana, which is the focus of this research, is government funded, receiving 0.5% of the overall health budget, or about 0.007% of gross domestic product (17, 18). In 2007, the WHO estimated that of the 21.6 million people living in Ghana, 650,000 were suffering from a severe mental disorder and a further 2,166,000 were suffering from a moderate-to-mild mental disorder (19). With only 32,283 people receiving treatment in 2007, the WHO further estimated that the treatment gap was 98% of the total population expected to have a mental disorder (19). In 2008, mental health services in Ghana were provided at only 68 health facilities and most of these were in the south of the country. These comprised 56 clinics run by community psychiatry nurses (CPNs); 5 regional hospital psychiatry units with a total bed capacity of 77 (located at Ho, Koforidua, Kumasi, Sunyani, and Wa); 3 state-owned psychiatric hospitals in Accra, Pantang, and Ankaful with a total bed capacity of 1,550; and 4 private psychiatric hospitals. The three psychiatric hospitals in Accra, Pantang, and Ankaful provided a total of 7.04 beds per 100,000 of the population (20). In 2011, Ghana had 11 psychiatrists in active service providing for the mental health needs of a population of nearly 25 million people (21). Because of the lack of adequate numbers of psychiatrists, the country's approximately 900 CPNs performed most direct mental health care (16). For several decades, CPNs have been trained in 3-year programmes at the Ankaful Nursing Training College located in the Central region of Ghana and at the Pantang Nursing Training School located in the Greater Accra region.

To address the human resource gap within Ghana's mental health delivery system, in March 2007, a link was established between Hampshire Partnership NHS Trust in the United Kingdom and the Rural Health Training School at Kintampo (RHTS), now designated as the College of Health and Well-Being, Kintampo (CoHK), in the Brong Ahafo region of Ghana. The link was initiated by the Tropical Health Education Trust to help improve mental health services in Ghana through the development of training curricula at RHTS. In November 2007, Ghana's Ministry of Health decided to create two new community-based mental health posts and develop curricula at RHTS to support these. The two posts were that of the medical assistant psychiatry (MAP) and the community mental health officer (CMHO), and it was envisaged that there would eventually be approximately one MAP and three or more CMHOs per district to compliment the work of CPNs and psychiatrists (22). The curricula for these new posts were developed by an expert group of mental health professionals from participating institutions in Ghana and the United Kingdom.

The designation MAP was later changed to ‘clinical psychiatry officer’ (CPO) (23). The CPO programme started in April 2010 with a first intake of nine students at the CoHK. Practicing CPOs are supervised by medical officers and psychiatrists (24).

In September 2011, the first cohort of 72 CMHOs graduated and started working across some of Ghana's 275 districts, taking mental health services to rural and underserved communities. Another 109 additional CMHOs graduated in September 2012 bringing the number of CMHOs working in Ghana as of 2013 to 181 (25). CPOs live and work in rural areas at the community level. They work in a system called Community-Based Health Planning and Services (CHPS). In CHPS, community health officers and community members are actively engaged as partners in the delivery of primary health care and family planning services.

The admission requirements, training duration, contents of the training curriculum, qualifications awarded, and clinical duties for CPNs, CPOs, and CMHOs are detailed in Table 1.

**Table 1:** Summary of training and clinical duties for the community mental health workers

Training and clinical responsibilities	Community mental health workers (CMHWs)		

	Community mental health officers (CMHOs)	Clinical psychiatric officers (CPOs)	Community psychiatric nurses (CPNs)
Admission requirement to training institution	Certificate in Community Health (or its equivalent from a recognized health training institution) or the possession of a Certificate in Community Health Nursing (or its equivalent from a recognized health training institution) plus a minimum professional service of 2 years in deprived areas or 3 years in non-deprived areas.	Advanced Diploma in Community Medicine and Health or an Advanced Diploma in Community Oral Health and Medicine plus a minimum professional service of 2 years in deprived areas or 3 years in non-deprived areas.	West African Senior School Certificate Examinations, the Senior Secondary School Certificate Examination or equivalent high school diplomas.
Specialized mental health training	Duration of Course: 12 months full-time training Curriculum content: General psychiatry and psychology, Community mental health practice, drugs, medication, physical health and psychiatry, team building and communication, ethics and professionalism, research, audit and evidence-based practice, basic clinical psychopharmacology, psychosocial aspects of treatment, child psychiatry, learning disability, and older peoples mental health (26). Qualification awarded: Diploma in Community Mental Health	Duration of course: 24 months full-time training plus 1–3 months ‘housemanship’ in specialist hospitals Curriculum content: General psychiatry; psychosocial perspectives of psychiatric practice; community mental health practice; leadership; team building and communication; managing change, supervision, and appraisal; ethics and professionalism; research and evidence-based practice; psychopharmacology; psychosocial intervention; history of psychiatry in Ghana; psychiatry and the law. Epilepsy, substance misuse psychiatry, liaison psychiatry, child psychiatry, learning disability and rehabilitation (23). Qualification awarded: Degree in Community Medicine and Clinical Psychiatry	Duration of course: 36 months full-time training *(first 18 months involves general nursing training, and the second 18 months is devoted to mental health nursing)* plus working as clinical mental health nurses for 2 years, and then in-service training of up to 3 months under an experienced CPN Curriculum content: Anatomy, physiology, pharmacology, obstetrics and gynecology, medicine, general nursing, psychology, psychopharmacology, clinical psychiatry Qualification awarded: Diploma in Nursing
Mental health clinical duties	- Conduct house-to-house visits to detect cases of mental disorder - Provide psychosocial support, such as family support and education, informal counseling and rehabilitation - Monitoring and supervision of patient's adherence to treatment (case management) - Refer cases to CPNs, CPOs, and other appropriate health professionals - Produce reliable data on mental disorders at the community level	- Have clearly delineated guidelines on which disorders to manage and which to refer - They have clear protocols for treatment and a limited range of drugs for prescribing - They are not be able to diagnose detailed subcategories of conditions outside those they treat (e.g. they might recognize anxiety disorder but would not differentiate generalized anxiety disorder from panic disorder; they might recognize personality disorder but they would not subtype it)	- Case detection and case management within the community, including monitoring patients’ compliance with psychotropic medication. - Case referral to District Medical Officers and psychiatrists.
	- Organize mental health promotion activities at the community level - Ensure community participation to promote mental health - Develop effective professional relationships with clients and their families - Do not prescribe medication or carry out any other independent form of treatment (26).		

The bulk of modern mental health care at the community level in Ghana is therefore currently provided by CMHOs, CPOs, and CPNs who are all government employees on the payroll of the Ministry of Health. It was envisaged that qualification as a CPO or CMHO would offer the health worker not only specialized duties but also enhanced remuneration, although according to the chief executive officer of the newly established Ghana Mental Health Authority, the salary structures for these health cadres are yet to be agreed upon. It is now 3 years since the first cohort of CMHOs, and 2 years since the first cohort of CPOs started working in the health system, and this study aims to generate new knowledge about the actual duties being performed by community mental health workers (CMHWs), including those that fall outside their job description and also explore how well integrated into the mental health system they are by assessing what proportion of the CMHWs are working closely with other health professionals and traditional healers. The specific research questions included

1. What proportion of CMHWs works closely with other health professionals and how frequently do the CMHWs collaborate with traditional healers?
2. How confident are CMHWs in their ability to recognize and treat specific mental health conditions, and which are the most frequently treated conditions?
3. How frequently do CMHWs prescribe medication, provide counseling, and visit patients in their homes?
4. What other duties do CMHWs routinely perform and which of these duties fall outside their job description?
5. Do the CMHWs perceive that they experience burnout from the work they do?

Finding answers to these questions will enable us to identify some of the problems or gaps that exist in the provision of mental health services at the community level in Ghana and make recommendations to improve the practice of CMHWs. We hypothesized that the role currently played by CMHWs would be influenced not only by their training and job description but also by the mental health needs of people in their communities and the availability of other community mental health, allied health personnel, and psychiatrists to meet these needs in a collaborative way, as illustrated in Fig. 1. Consequently, we envisaged that some CMHWs would be working beyond their scope of training and formal job description in an effort to meet some of these needs. We also envisaged that these efforts increase the volume of work performed by the CMHWs and lead to high levels of burnout.

**Fig. 1:**
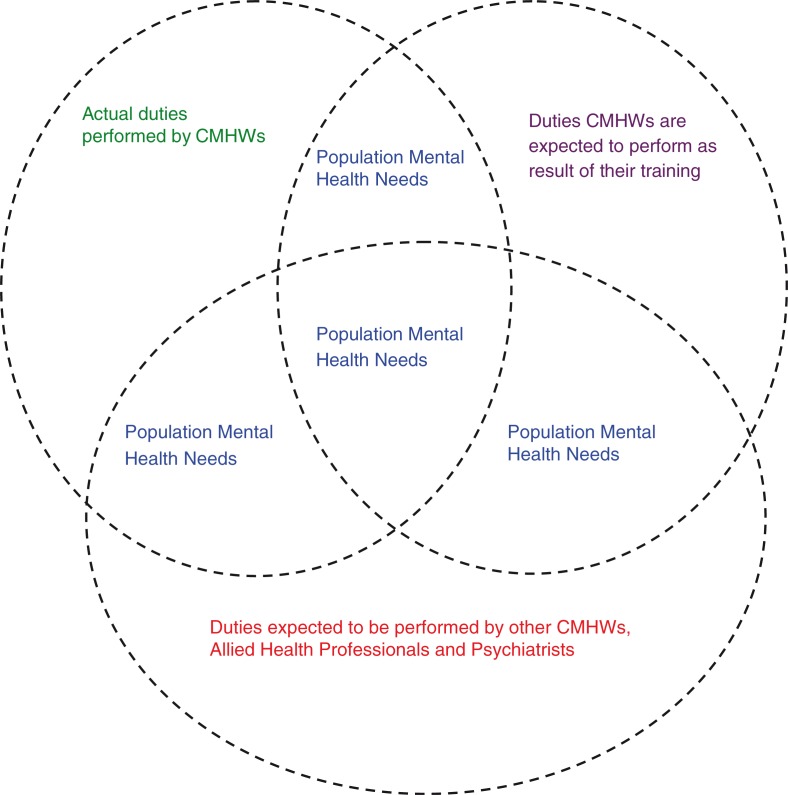
Conceptual framework underlining the role and scope of practice of CMHWs in Ghana.

## Methodology

The study design was a cross-sectional survey with mixed quantitative and qualitative methods. Purposive sampling was used to select CMHW respondents from all the 10 regions of Ghana so as to ensure regional balance in the responses, whereas convenience sampling approach was used for data collection from CMHWs within each region because of the geographical spread of these respondents.

A self-administered, semi-structured, 53-item questionnaire with optional answers, including Likert scales and open-ended questions, was developed by the research team based on a review of the literature on similar studies on task shifting using community health workers. The questionnaire was pretested on two respondents in each category of CMHWs, namely CPNs, CPOs, and CMHOs. They were revised based on the results of the pretest to give it a better structure and to introduce more Likert scales for some responses before being administered to the respondents. They generally took 10–15 min to complete and no monetary or other incentives were provided to the respondents. One section of the questionnaire containing 15 items (Supplementary file) assessed the role and scope of practice of the CMHWs which is the focus of this paper. This section of the questionnaire contained questions exploring the professionals the CMHWs work closely with; the level of confidence of the CMHWs in independently diagnosing and treating named mental disorders; a ranking of the frequency with which they treat the various mental conditions; and the frequency with which they provide counseling, prescribe medication, visit patients in their homes, and collaborate with traditional healers. It also contained questions about what other duties they perform routinely as part of their job description as well as the duties they routinely perform that fall outside their job descriptions.

The study received prior institutional review board approvals from the Health Policy and Management and Global Health Ethics Committee at Trinity College Dublin (Reference number: 07/2013/06) and the School of Medical Sciences, Kwame Nkrumah University of Science and Technology, Kumasi, Ghana (Reference number: CHRPE/AP/300/13). The study also received the approval of the office of the chief psychiatrists of the Ghana Health Service. All study participants received information leaflets about the study prior to completing the questionnaires. Written informed consent was obtained from each participant before they completed the survey questionnaires.

Data were collected between 10 August 2013 and 30 October 2013. Data from 164 CMHWs nationwide were obtained with the assistance of the national coordinator of the CPNs and his or her 10 regional representatives, as well as a CPO who works as a lecturer at the CoHK. The former initially distributed and returned all 100 questionnaires given to CMHWs including CPNs, CPOs, and CMHOs working in all 10 regions (10 questionnaires per region) through his or her regional coordinators. Nine additional questionnaires were photocopied, completed, and returned from some regions, bringing the number of completed questionnaires collected through the national coordinator for CPNs to 109. The latter distributed the questionnaires to 80 CPOs and CMHOs attending a conference at the college in August 2013 from which 55 questionnaires were returned. The 80 CPOs and CMHOs were among the group attending the conference who had not already completed the questionnaire through the regional coordinator of the CPNs. The overall response rate for all CMHWs was therefore 86.77%. Quantitative data from categorical variables including Likert scales were analyzed using descriptive and inferential statistics with SPSS version 20. We compared some of the responses offered by the different CMHWs using chi-square and Fisher's exact tests. Qualitative data compiled from responses to the open-ended questions on the survey questionnaire were subjected to a thematic analysis, with coding being performed manually.

## Results

### Characteristics of respondents

Overall, 164 mental health workers from all the 10 regions of Ghana, comprising 71 (43.3%) CPNs, 19 (11.6%) CPOs, and 74 (45.1%) CMHOs participated in the survey. The CMHWs had been working between 1 and 40 years in the domain of mental health with a mean of 4.7 years and a standard deviation (SD) of 6.98 years.

The CMHWs were asked to indicate which health-care workers they worked closely with. A chi-square/Fisher's exact test was used to compare the association between the CMHW types and the health-care workers they worked closely with. The results of this comparison are shown in Table 2.

**Table 2:** Comparison of the CMHW types and the health-care professionals they work closely with^a^

Health-care professionals	CMHW	*N* (%)	df	Chi-square (*X* ^2^)/Fisher's exact test^a^	*P*
Psychiatrists	CMHO	3 (4.1)	2	a	0.00
	CPO	7 (36.8)			
	CPN	29 (40.8)			
	Total *N* (%)	39 (23.8)			
CPNs	CMHO	50 (67.6)	2	a	0.01
	CPO	16 (84.2)			
	CPN	62 (87.3)			
	Total *N* (%)	128 (78)			
CMHOs	CMHO	49 (66.2)	2	a	0.29
	CPO	12 (63.2)			
	CPN	38 (53.8)			
	Total *N* (%)	99 (60.4)			
CPOs	CMHO	17 (23)	2	14.34	0.01
	CPO	13 (68.4)			
	CPN	27 (38)			
	Total *N* (%)	57 (34.8)			
Social workers	CMHO	21 (28.4)	2	7.46	0.02
	CPO	11 (57.9)			
	CPN	32 (45.1)			
	Total *N* (%)	64 (39)			
Psychologists	CMHO	11 (14.9)	2	11.66	0.00
	CPO	7 (36.8)			
	CPN	28 (39.4)			
	Total *N* (%)	46 (28)			
Occupational therapists	CMHO	3 (4.1)	2	a	0.25
	CPO	2 (10.5)			
	CPN	8 (11.3)			
	Total *N* (%)	13 (7.9)			
Other colleagues	CMHO	7 (9.5)	2	a	0.44
	CPO	1 (5.3)			
	CPN	3 (4.2)			
	Total *N* (%)	11 (6.7)			

a The percentage for each CMHW type is based on the total number of respondents within the CMHW type which was 71 for CPNs, 19 for CPOs, and 74 for CMHOs. Similarly, the ‘Total N’ is based on the total number of CMHW respondents which was 164.

The results in Table 1 indicate that there was a significant difference between the CMHW types and those working closely with psychiatrists, CPNs, CPOs, social workers, and psychologists, but not occupational therapists and CMHOs. The results indicate, for example, that significantly fewer CMHOs worked closely with psychiatrists, psychologists, and social workers compared with CPOs and CPNs.

### Diagnosing and treating mental disorders – are CMHWs confident in their abilities?

Nearly all CMHWs (98.6% of CMHOs, 100% of CPOs, and 98.6% of CPNs) expressed confidence in their ability to assess and diagnose mental health conditions in general. The results of confidence in diagnosing specific mental health conditions are shown and compared in Table 3.

**Table 3:** Comparison of the CMHW types and their expression of confidence about their ability to diagnose specific mental conditions^a^

Mental disorder	CMHW	*N* (%)	df	Chi-square (*X* ^2^)/Fisher's exact test^a^	*P*
Schizophrenia	CMHO	63 (85.1)	2	a	0.54
	CPO	18 (94.7)			
	CPN	62 (87.3)			
	Total *N* (%)	143 (87.2)			
Mood disorders	CMHO	7 (95.9)	2	a	0.41
	CPO	19 (100)			
	CPN	63 (93)			
	Total *N* (%)	156 (95.1)			
Anxiety disorders	CMHO	46 (62.2)	2	a	0.08
	CPO	18 (94.7)			
	CPN	49 (69)			
	Total *N* (%)	113 (68.9)			
Eating disorders	CMHO	26 (35.1)	2	2.58	0.28
	CPO	10 (52.6)			
	CPN	32 (45.1)			
	Total *N* (%)	68 (41.5)			
Personality disorders	CMHO	22 (29.7)	2	8.24	0.02
	CPO	12 (63.2)			
	CPN	23 (45.1)			
	Total *N* (%)	66 (40.2)			
Addiction disorders	CMHO	60 (81.1)	2	a	0.10
	CPO	18 (94.7)			
	CPN	65 (91.5)			
	Total *N* (%)	143 (87.2)			
Child and adolescent disorders	CMHO	31 (41.9)	2	5.19	0.08
	CPO	13 (68.4)			
	CPN	39 (54.9)			
	Total *N* (%)	83 (50.6)			
Other mental disorders	CMHO	28 (37.8)	2	0.67	0.70
	CPO	9 (47.4)			
	CPN	30 (42.3)			
	Total *N* (%)	67 (40.9)			

a The percentage for each CMHW type is based on the total number of respondents within the CMHW type which was 71 for CPNs, 19 for CPOs, and 74 for CMHOs. Similarly, the ‘Total N’ is based on the total number of CMHW respondents which was 164.

There was no significant difference in the proportion of the different CMHW types who expressed confidence in their ability to diagnose any of the named mental health conditions except personality disorders. For personality disorders, a significantly higher proportion of CPOs expressed confidence in their ability to diagnose compared with CPNs and CMHOs. The results on confidence of CPOs in treating specific mental health conditions are shown in Table 4.

**Table 4:** Comparison of the CMHW types and an expression of confidence about their ability to treat specific mental conditions^a^

Mental disorder	CMHW	*N* (%)	df	Chi-square (*X*^2^)/Fisher's exact test^a^	*P*
Schizophrenia	CMHO	60 (81.1)	2	a	0.00
	CPO	19 (100)			
	CPN	68 (95.8)			
	Total *N* (%)	147 (89.6)			
Mood disorders	CMHO	59 (79.7)	2	a	0.01
	CPO	18 (94.4)			
	CPN	68 (95.8)			
	Total *N* (%)	145 (88.4)			
Anxiety disorders	CMHO	38 (51.4)	2	a	0.01
	CPO	16 (84.2)			
	CPN	50 (70.4)			
	Total *N* (%)	104 (63.4)			
Eating disorders	CMHO	10 (13.5)	2	6.10	0.05
	CPO	7 (36.8)			
	CPN	18 (25.4)			
	Total *N* (%)	35 (21.3)			
Personality disorders	CMHO	9 (12.2)	2	20.97	0.00
	CPO	10 (52.6)			
	CPN	30 (42.3)			
	Total *N* (%)	49 (29.9)			
Addiction disorders	CMHO	45 (60.8)	2	a	0.00
	CPO	18 (94.7)			
	CPN	66 (93.0)			
	Total *N* (%)	129 (78.7)			
Child and adolescent disorders	CMHO	24 (32.4)	2	8.46	0.02
	CPO	9 (47.4)			
	CPN	40 (56.3)			
	Total *N* (%)	73 (44.5)			
Other mental disorders	CMHO	34 (45.9)	2	0.49	0.78
	CPO	9 (47.4)			
	CPN	29 (40.8)			
	Total *N* (%)	72 (43.9)			

a The percentage for each CMHW type is based on the total number of respondents within the CMHW type which was 71 for CPNs, 19 for CPOs, and 74 for CMHOs. Similarly, the ‘Total N’ is based on the total number of CMHW respondents which was 164.

Unlike with diagnosis, there was a statistically significant difference in the confidence expressed by the different CMHW groups in their ability to treat all the named mental health conditions except other conditions. A significantly higher proportion of CPOs than CMHOs and CPNs expressed confidence in their ability to treat all the disorders except mood disorders and child and adolescent disorders which had a relatively higher proportion of CPNs saying they were confident about treating than CPOs and CMHOs.

### Scope of work for the CMHWs

The mental health workers were asked to rank on a scale of 1 to 8 which mental health conditions they treat frequently, where 8 meant most frequently treated and 1 meant least frequently treated. Table 5 indicates the means and SD of the various numbers indicted for the various conditions.

**Table 5:** Mean and SD of the of the numerical scale^a^ of 1 to 8 of frequencies of the conditions commonly treated by all the CMHWs

	Schizophrenia	Mood disorders	Anxiety disorders	Eating disorders	Personality disorders	Addiction disorders	Child mental disorders	Other disorders
Mean (SD)	7.07 (1.29)	6.13 (1.59)	4.80 (1.76)	2.37 (1.67)	3.05 (1.70)	5.71 (1.73)	3.47 (1.92)	6.65 (2.24)

a 1 indicates the condition they treat least frequently and 8 indicates the condition they treat most frequently.

Table 5 indicates that schizophrenia was the condition most frequently treated by the mental health workers followed by other conditions, and then mood disorders. Eating disorders were the conditions that were least frequently treated by the mental health workers. Other conditions treated by the CMHWs included epilepsy (87 CMHWs), puerperal psychosis (18 CMHWs), migraines (9 CMHWs), bed wetting (8 CMHWs), dementia (5 CMHWs), sleep disorders (3 CMHWs), and sexual disorders (2 CMHWs).

A one-way, between-group analysis of variance was conducted to explore the impact of CMHW type on the frequency with which each of the specified mental health disorders were reported to be treated. There was no statistically significant difference in the reported frequency with which the three groups of CMHWs treated any of the mental health conditions, as shown in Table 6.

**Table 6:** An analysis of variance (ANOVA) comparing the rankings for the various mental disorders treated by the CMHWs

Mental disorder	CMHW	*N*	Mean (on a scale of 1 to 8)	Standard deviation (SD)	Degree of freedom (df) between groups	Mean square	*F*	*P*
Schizophrenia	CMHO	65	6.78	1.57	2	4.819	2.956	0.055
	CPO	18	7.17	0.92				
	CPN	70	7.31	1.03				
Mood disorders	CMHO	63	6.06	1.57	2	0.338	0.132	0.877
	CPO	18	6.28	1.71				
	CPN	71	6.14	1.60				
Anxiety disorders	CMHO	55	5.16	1.82	2	6.087	1.987	0.141
	CPO	19	4.50	2.13				
	CPN	65	4.57	1.58				
Eating disorders	CMHO	41	2.83	2.04	2	7.285	2.670	0.074
	CPO	12	2.08	1.83				
	CPN	51	2.06	1.21				
Personality disorders	CMHO	41	2.76	1.88	2	4.173	1.440	0.241
	CPO	15	3.60	1.72				
	CPN	60	3.12	1.56				
Addiction disorders	CMHO	59	5.39	1.77	2	5.247	1.755	0.177
	CPO	18	5.89	1.94				
	CPN	71	5.94	1.64				
Child and adolescent disorders	CMHO	43	3.39	1.89	2	2.040	0.547	0.580
	CPO	15	3.07	1.71				
	CPN	63	3.62	2.00				
Other mental disorders	CMHO	41	6.98	1.98	2	5.390	1.074	0.347
	CPO	9	5.89	2.42				
	CPN	31	6.45	2.50				

Post-hoc comparisons using the Tukey honest significant difference test indicated that the mean score for CPNs (*M=*7.31, SD=1.03) was significantly different from CMHOs (*M=*6.78, SD=1.57) for only schizophrenia. None of the other comparisons produced a statistically significant difference in mean scores for any specified mental health disorder.

**Fig. 2:**
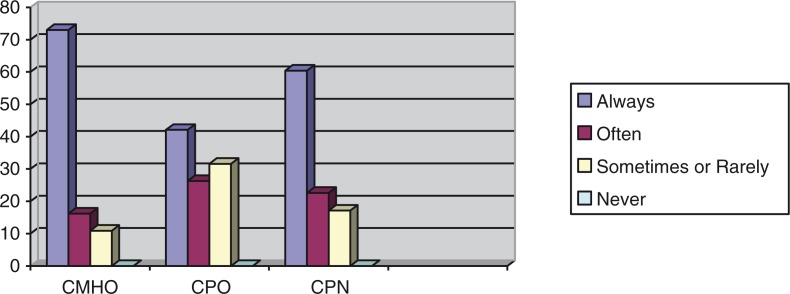
Percentages of the CMHW groups and the frequency with which they provide counseling (df=8, *p=*0.05).

**Fig. 3:**
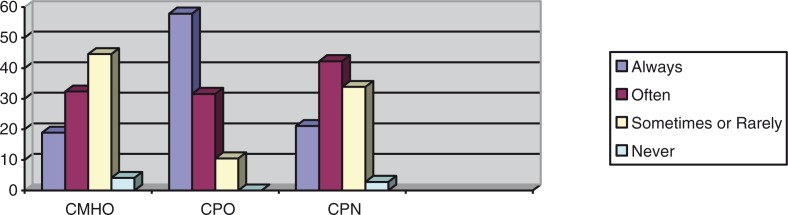
Percentages of the CMHW groups and the frequency with which they prescribe medication (df=8, *p=*0.02).

**Fig. 4:**
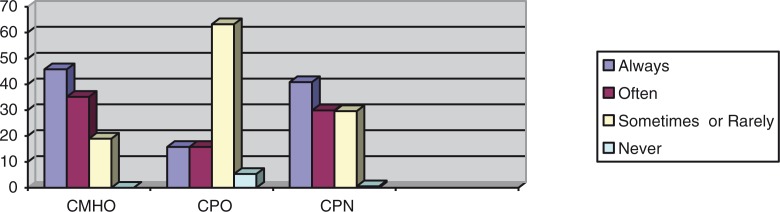
Percentages of the CMHW groups and the frequency with which they visit patients in their homes (df=8, *p=*0.00).

Frequencies with which the CMHWs provide counseling, prescribe medication, and visit patients in their homes were compared using Fisher's exact test, and these showed statistically significant associations between the CMHW type and the frequency with which they perform tasks, as shown in Figs. 2–4.

The above results indicate that there was a statistically significant difference between the different CMHW types and the frequency with which they said they provide counseling, prescribe medication, or visit patients in their homes. Figure 2 suggests that a significantly higher proportion of CMHOs (73%) reported that they always provide counseling compared with CPOs (42.1%) and CPNs (51.2%). Figure 3 also suggests that a significantly higher proportion of CPOs (57.9%) said that they always prescribe medication, compared with CMHOs (18.9%) and CPNs (21.1%). Of interest, 98.6% of CMHOs reported that they always, often, or sometimes prescribe medication. Fig. 4 also indicates that a significantly higher proportion of CMHOs (80.9%) reported that they always or often visit patients in their homes compared with CPOs (31.6%).

All three groups of CMHWs identified additional tasks which form part of their routine duties within the districts in which they work, including health education in hospital outpatient departments, schools, churches, and community durbars (gathering of chiefs and their folks). Other tasks they said they perform include reproductive and child health services; training herbalists, spiritualists, and church leaders involved in mental health activities; consultation–liaison psychiatric services in district hospitals; and advocating on behalf of patients and their relatives or linking them with social services. They also said they write reports and carry out other administrative functions including liaising with stakeholders such as voluntary organizations and the district administrative authorities on mental health issues within the district. In addition, some CMHOs and CPNs reported that they trace the homes of mental health patients and also follow up on those who default in attending appointments for review or medication. They also said they follow district medical officers during ward rounds in general hospitals to identify mental health cases.

When asked to speculate if patients they care for would have received any form of mental health care, assuming that they were not in your current job, 27% of CMHOs, 36.8% of CPOs, and 21.1% of CPNs answered ‘unlikely’, with the rest answering ‘likely’. A chi-square test for independence indicated no significant association between the CMHW type and the response provided, *X*^2^ (2, *n=*164)=2.08, *p=*0.35.

Overall, 90.5% of CMHOs, 100% of CPOs, and 100% of CPNs said they clearly understood their job description, whereas the remaining 9.5% of CMHOs said they did not have a clear understanding of their job description. Overall, 15 (9.1%) CMHWs said they always performed tasks outside their job description, 37 (22.6%) said they often performed tasks outside their job description, 79 (48.2%) said they sometimes performed tasks outside their job description, 20 (12.2%) said they rarely perform tasks outside their job description, and only 13 (7.9%) said they never performed tasks outside their job description. A comparison of the frequency with which the different CMHWs performed tasks outside their job description using Fisher's exact test showed no statistically significant association between the CMHW types.

Tasks performed by the CMHOs and CPNs which they perceived fell outside their job description included performing general and other specialized nursing duties such as dressing wounds; providing antenatal, family planning, and immunization services; treating physical ailments; diagnosing mental conditions; prescribing medication; and reviewing the condition of mental health patients in district hospital wards. One CMHO wrote

One day a patient was brought to the facility with an epileptic attack by a mental health nurse and the in-charge was not around so myself and one other colleague quickly gave IV Diazepam so as to abort the seizure even though in actual fact, a CMHO is not supposed to do so.

Another CMHO wrote: ‘my job description says I should assist in treating but I assess and treat every case since the CPN died’.

Tasks performed by the CPOs which they perceived fell outside their job description included undertaking liaison psychiatric consultations in regional hospitals and undertaking general medical consultations. One CPO wrote: ‘I have been assisting at the medical outpatient department to care for accident cases and other emergencies’.

All three groups of CMHWs said they have had to provide financial assistance to their patients by offering to pay for their medication, food, or transportation. They all said they have had to advocate for social services on behalf of patients.

Overall, 62 (83.8%) CMHOs, 18 (94.7%) CPOs, and 65 (91.5%) CPNs reported that there are clear treatment protocols which guide their work. The remaining CMHWs denied knowledge of any such treatment protocols.

Furthermore, 69 (93.2%) CMHOs, 17 (89.5%) CPOs, and 68 (95.8%) CPNs responded that they cope, at least to some extent, with the volume of work that they do on a day-to-day basis. However, 59 (79.7%) CMHOs, 13 (68.4%) CPOs, and 57 (80.3%) CPNs reported ‘burnout’ from their work. A chi-square test for independence indicated no significant association between the CMHW types and whether or not they experienced burnout.

**Fig. 5:**
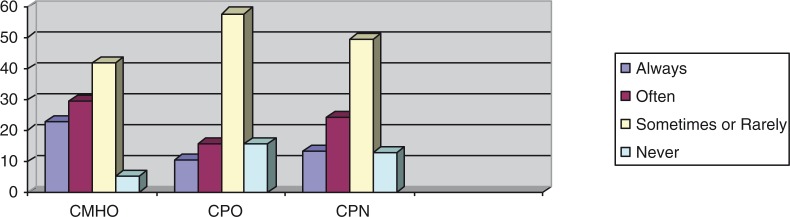
Percentages of the CMHW groups and the frequency with which they collaborate with traditional healers (df=8, *p=*0.00).

A comparison of the frequency with which the CMHWs collaborate with traditional healers was made, which showed a statistically significant association between the CMHW types and the frequency with which they collaborate with traditional healers, as shown in Fig. 5.

The results in Fig. 5 indicate that a significantly higher proportion of CMHOs (52.7%) reported that they always or often collaborate with traditional healers compared with CPOs (26.3%) and CPNs (25.3%). Overall, 75% of all CMHWs reported that they always, often, or sometimes collaborated with traditional healers.

## Discussion

Our results suggest that less than a quarter of all CMHWs work closely with a psychiatrist. This is not surprising given that there were only 18 psychiatrists with only 11 of them actively practicing in Ghana in 2011 (27). Our results also suggest that far fewer than half of all CMHWs work closely with a social worker, psychologist, or occupational therapist. These statistics are also not surprising given that in 2011, there were only 19 clinical psychologists (0.08 per 100,000), 21 social workers (0.09 per 100,000), and 4 occupational therapists (0.02 per 100,000) working within Ghana's mental health delivery system (27). The landmark *Lancet* global mental health series of 2007 and 2011, along with WHO's *Atlas: Mental Health Resources in the World*, also highlighted the major shortages of psychiatrists, psychiatric nurses, psychologists, and social workers in LAMICs (7, 28, 29). Our study did not explore the impact of collaboration between CMHWs and various health workers on the productivity or efficacy of the CMHW. However, we see this as an important area for future study. As a result of the shortage of higher level mental health cadres, including psychiatrists, all CMHWs in Ghana perform tasks outside their formal job descriptions and level of training to meet both the physical and mental health needs of patients in their communities. To alleviate the health worker crisis in Ghana as in most parts of sub-Saharan Africa, there is therefore the need to revise the training and scope of practice of the CMHWs and to remove some of the barriers to professional practice that exist. Mental state examination, diagnosis, and treatment of common mental disorders should not be the preserve of psychiatrists alone in a resource-poor country. Certain functions need to be assumed by lower-skilled cadres of health workers who are offered appropriate training to achieve a more sustainable skills match for wider health worker coverage (3). The current situation is that many of these cadres are put in a position where they feel pressured into working beyond their scope of practice to respond to community needs. This is unsatisfactory for all stakeholders as it involves risks for both the patient and for the CMHWs, and if misdiagnoses or inappropriate care occurs, it may also have implications for more senior staff in the health services. It also has negative implications for the referral system as lower-skilled cadres not working closely with psychiatrists, psychologists, social workers, or occupational therapists means the ease of referral for psychiatric and other types of consultations and opportunities for multidisciplinary working are critical missing elements in the system. Pressing health needs across the globe cannot be met without adequate numbers of well-trained and available health workers (3). Given the results of our study, it may be useful for the training of CMHWs in Ghana to not only focus on nursing and medical care but also to include other areas such as the provision of social and psychological care. The health workforce is the backbone of each health system; the lubricant that facilitates the smooth implementation of health action for sustainable socioeconomic development (3).

Of special significance, about half of all CMHOs compared to only a quarter each of all CPOs and CPNs reported that they always or often collaborated with traditional healers. This is expected as a higher proportion of CMHOs work mainly in remote communities where traditional healers usually operated. It is expected that such collaboration will bring mental health care to many who otherwise would have had no access to conventional psychiatric treatments. In a situational analysis of the challenges and opportunities at the district level of five LAMICs implementing integrated mental health care, it was reported that across all sites, community mental health literacy was low and there were no models of multisectoral working or collaborations with traditional or religious healers (30).

Our results show that all the CMHW categories expressed similar levels of confidence in independently diagnosing all mental health conditions except personality disorders. As expected, however, a significantly smaller proportion of CMHOs expressed confidence in their ability to independently treat all the named mental health conditions compared with CPOs and CPNs. What seems surprising is that some CMHOs who are the least trained mental health cadres and whose core duties exclude treatment of mental conditions seem to be fairly confident about their ability to independently treat these disorders. Furthermore, these results suggest that the training offered to CPOs may be appropriate in preparing them for the role of treating common mental conditions which forms part of the core duties of CPOs. Similarly, although unofficial, CPNs have been treating patients in remote districts of Ghana for several decades because of the absence of adequate numbers of trained psychiatrists, and our results suggest that the training offered to them may prepare them for this role. Interestingly, our results suggest that there is no significant difference among CMHOs, CPOs, and CPNs with regard to the mental health conditions that they frequently treat independently. This is worrying, as the core training for CMHOs includes case detection and case monitoring, but not independent case treatment. Furthermore, because CMHOs reported significantly less confidence in their ability to treat all the named mental health conditions, they are least expected to report that they independently treat mental health patients at the same frequency as CPOs and CPNs. Furthermore, the CMHWs reported schizophrenia as the most frequently treated mental health condition and there was no statistically significant difference in the reported frequency with which the three groups of CMHWs treated any of the mental health conditions. This suggests that all three groups of CMHWs appear to perform similar roles with regard to treatment of mental health conditions, and this raises concerns about the quality of the care patients receive, given that there are marked differences in their training and expectations about their roles within the mental health delivery system. It will therefore be necessary for mental health policy makers in Ghana and the mental health training institutions to critically examine the role of all the CMHWs, in particular, the role of CMHOs, to ensure that they are appropriately trained to fulfill the added roles which they assume on the field.

As expected, most CMHWs reported that they always or often offer counseling to their patients even though a significantly larger proportion of CMHOs than CPOs and CPNs reported that they always offer counseling to their patients. Again, a significantly higher proportion of CMHOs than CPOs and CPNs reported that they visit patients in their homes which is consistent with the provisions of their core training and job descriptions. Furthermore, a significantly higher proportion of CPOs reported that they always or often prescribe medication compared with CMHOs and CPNs. This is also expected because the training of CPOs equips them with the knowledge and skills to prescribe some basic psychotropic medication. On the contrary, about 40% of CMHOs reported unexpectedly that they always or often prescribe medication contrary to provisions of their core training and job description, which should be a source of concern for policy makers and service coordinators. Also, the fact that not all of the CMHOs and CPNs reported that they always or often offer counseling or visit patients in their homes raises questions about whether some of these CMHWs may be neglecting some of their core responsibilities while taking on added roles outside of their core duties.

A large proportion of all the three categories of CMHWs perceived that they perform duties outside of their job description and level of formal training. This is not surprising, given that most of them do not work in conjunction with other allied mental health professionals but are expected to meet the needs of patients. This situation is comparable with what pertains in Uganda, for example, where there has been a gradual shift of the task to set up intravenous lines from doctors to nurses which is not protected by existing laws and regulations for that country (31). The duties performed by CMHWs which they said fall outside their job description included dressing of wounds; providing antenatal, family planning, and immunization services; and also treating physical ailments. These greatly expand the role and scope of practice for CMHWs in Ghana which has the potential to compromise the quality of mental health care and lead to burnout, as many CMHWs find themselves working alone in remote districts without formal supervision arrangements, and with inadequate logistic and technical support from district health management teams. To ensure adequate quality, reduce burnout, and encourage recognition of responsibilities, the provision of training, supervision, monitoring, and evaluation for health-care workers are all critical (32). It is important for mental health policy makers to stress to lesser trained health cadres the importance of recognizing their limitations and to seek to refer patients in situations where the patients’ mental or physical health issues are beyond what they can manage based on their training and experience. Alternatively, CMHWs should either be provided with the necessary training to enable them to fulfill these additional roles, which should then be incorporated into their official duties, or other community health workers should be urgently trained and deployed across the various districts in sufficient numbers to offer these specialized services, so that CMHWs can focus on delivering on their core mandates. It will be interesting to know if other specialized community health workers in Ghana, such as community midwives, routinely perform duties outside their job description, so that appropriate conclusions can be drawn regarding discrimination against the CMHWs. For example, do community midwives in Ghana also provide mental health care for patients attending antenatal services? These may be the focus of further research arising out of this study.

A limitation of our study is that although the survey questionnaire was well researched based on the literature from previous studies in the field and pretested before use, it was nonetheless not a validated instrument. Furthermore, the paper and pen approach adopted in the survey might have limited the responses of the respondents to some of the open-ended questions, the net results of which has been that the results are predominantly quantitative in nature with limited qualitative information. Another limitation was the small sample size for CPOs which hindered prospects for further statistical exploration of the responses according to the CMHW type. These limitations notwithstanding, a nationally representative sample of CMHWs were included, something which we consider a strength of this study.

## Conclusions

Our study highlights several important gaps in the service provision at the community level within Ghana's mental health delivery system; however, the most of CMHWs surveyed expressed confidence in their ability to recognize and treat most of the common mental disorders. We have established through this study that only about a quarter of all CMHWs work closely with psychiatrists, psychologists, and social workers, and a similar proportion collaborate with traditional healers. We have also established that there are no statistically significant differences in the frequency with which the three groups of CMHWs treat any of the mental health conditions even though CMHOs are not expected to treat patients as part of their duties. Furthermore, we have established that in addition to duties prescribed in their job descriptions, all the CMHW groups perform several jobs for which they have no training including dressing wounds; providing antenatal, family planning, and immunization services; treating physical ailments; and prescribing medication in the case of CMHOs. Mental health policy makers and coordinators need to thoroughly review the training curriculum and also evaluate the job descriptions of all CMHWs in Ghana to ensure that they are consistent with the demands and health-care needs of patients they care for in their communities. For example, as CMHOs and CPNs prescribe medication even though they are not expected to do so, it may be worth exploring the merits of including the prescription of common psychotropic medication in their curriculum and also job descriptions.

## Authors’ information

VIOA is a Clinical Associate Professor at the Department of Psychiatry, University of Alberta, Canada. He is also a visiting faculty member at the Department of Behavioural Sciences, Kwame, Nkrumah University of Science and Technology, Ghana, and a research associate at the Centre for Global Health, University of Dublin, Trinity College Dublin, Ireland. AO is the Chief Executive Officer of the Ghana Mental Health Authority and the Chief Psychiatrist at the Accra Psychiatric Hospital in Accra, Ghana. CKF is a Clinical Associate Professor at the Department of Psychiatry, University of Dublin, Trinity College Dublin, Ireland. EM is the immediate past Director of the Centre for Global Health, University of Dublin, Trinity College Dublin, Ireland. She is currently a Professor and Chair of Health Systems at the School of Nursing, Midwifery and Health Systems, University College Dublin, Ireland.
